# Dysfunctional Inflammation in Cystic Fibrosis Airways: From Mechanisms to Novel Therapeutic Approaches

**DOI:** 10.3390/ijms22041952

**Published:** 2021-02-16

**Authors:** Alessandra Ghigo, Giulia Prono, Elisa Riccardi, Virginia De Rose

**Affiliations:** 1 Department of Molecular Biotechnology and Health Sciences, Molecular Biotechnology Center, University of Torino, 10126 Torino, Italy; giulia.prono@edu.unito.it; 2 Postgraduate School in Respiratory Medicine, University of Torino, 10126 Torino, Italy; elisa.riccardi@unito.it

**Keywords:** cystic fibrosis, inflammation, anti-inflammatory treatment, CFTR modulators

## Abstract

Cystic fibrosis (CF) is an inherited disorder caused by mutations in the gene encoding for the cystic fibrosis transmembrane conductance regulator (CFTR) protein, an ATP-gated chloride channel expressed on the apical surface of airway epithelial cells. CFTR absence/dysfunction results in defective ion transport and subsequent airway surface liquid dehydration that severely compromise the airway microenvironment. Noxious agents and pathogens are entrapped inside the abnormally thick mucus layer and establish a highly inflammatory environment, ultimately leading to lung damage. Since chronic airway inflammation plays a crucial role in CF pathophysiology, several studies have investigated the mechanisms responsible for the altered inflammatory/immune response that, in turn, exacerbates the epithelial dysfunction and infection susceptibility in CF patients. In this review, we address the evidence for a critical role of dysfunctional inflammation in lung damage in CF and discuss current therapeutic approaches targeting this condition, as well as potential new treatments that have been developed recently. Traditional therapeutic strategies have shown several limitations and limited clinical benefits. Therefore, many efforts have been made to develop alternative treatments and novel therapeutic approaches, and recent findings have identified new molecules as potential anti-inflammatory agents that may exert beneficial effects in CF patients. Furthermore, the potential anti-inflammatory properties of CFTR modulators, a class of drugs that directly target the molecular defect of CF, also will be critically reviewed. Finally, we also will discuss the possible impact of SARS-CoV-2 infection on CF patients, with a major focus on the consequences that the viral infection could have on the persistent inflammation in these patients.

## 1. Introduction

Cystic fibrosis (CF) is an inherited, life-threatening disorder caused by mutations in the gene encoding for the cystic fibrosis transmembrane conductance regulator (CFTR), an ATP-gated chloride (Cl-) channel expressed on the apical side of airway epithelial cells. Defective CFTR activity results in altered ion transport and the subsequent dehydration and hypersecretion of mucus that contributes to airway obstruction. Moreover, this abnormally thick and sticky mucus layer entraps noxious agents and pathogens that initiate a persistent and self-perpetuating inflammatory response. This latter is recognized as a major driver of the progressive structural damage of the airways and lung parenchyma in CF patients, ultimately leading to bronchiectasis and respiratory failure [1].

The inflammatory response in CF airways occurs early in the disease process. Increasing evidence suggests that CFTR dysfunction itself drives a dysregulated inflammatory response and that, before any infection, CF airways are already in a proinflammatory state that provides a fertile substrate for subsequent tissue damage and chronic infection [2,3,4]. When pathogens colonize the already-dysfunctional airway microenvironment, activation of host protective mechanisms, including release of proteases, reactive oxygen/nitrogen species (ROS/RNS), and proinflammatory chemokines by epithelial and inflammatory cells, is exaggerated, and may cause tissue damage [5]. Recognition of both pathogen-associated molecular patterns (PAMPs) and damage-associated molecular patterns (DAMPs) by cognate receptors on different immune cell subsets (neutrophils, macrophages, and dendritic cells) further contributes to the production of proinflammatory mediators, such as tumor necrosis factor-alpha (TNF-α), interleukin (IL)-1, and IL-6. In addition, several chemoattractants, including IL-8, lipids, and complement fragments, ensure the increase of vascular permeability and the recruitment of circulating leukocytes to the lung to sustain the inflammatory response [6].

Neutrophils represent the major immune cell type infiltrating the airways in CF. Once they reach the site of infection and upon local activation, neutrophils produce reactive oxygen species, proteinase 3, cathepsin G, and elastase, potent toxic agents that, when present in excess, can indiscriminately damage bacteria and host cells. The ensuing injury of healthy lung tissue exacerbates immune/inflammatory cell recruitment to the site of infection, as well as lung damage [7]. In addition, leukocyte infiltration into the lung, and the intertwined tissue damage, are further stimulated by small fragments of the extracellular matrix (such as elastin and collagen) that are the cleavage products of neutrophil-derived serine and matrix metalloproteases (MMPs) [8]. Of note, the peculiar excess of neutrophils in the CF lung is due not only to an increased influx, but also to impaired clearance. While in physiological conditions neutrophils undergo apoptosis and are cleared by alveolar macrophages [9,10], in CF they often experience necrosis, causing the release of intracellular contents, including chemoattractants (such as IL-8 and LTB4) that further fuel neutrophil influx. Therefore, despite being essential in antibacterial host defense, neutrophils and their products represent key drivers of a self-perpetuating cycle of inflammation [11].

Because an exaggerated inflammatory process plays a central role in the progression of airway-wall remodeling, lung damage, and lung-function decline in patients affected by CF [12], anti-inflammatory treatment would be a cornerstone in CF management. In fact, these therapies, combined with antibiotic therapy, offer a rational approach to prevent chronic lung damage in the disease. However, the inadequate efficacy and/or safety profiles of traditional anti-inflammatory drugs limit their clinical application. Moreover, despite approved CFTR modulators targeting the basic molecular defect of CF and holding great promise for the treatment of CF lung disease, their anti-inflammatory properties are still inconclusively demonstrated. Overall, these observations highlight the importance of investing efforts in the development of new effective and safe anti-inflammatory drugs, even in the age of CFTR modulators. This review article aims to provide an overview of anti-inflammatory therapies currently available or under development for the treatment of CF lung disease. Recent reports on the potential anti-inflammatory properties of CFTR modulators will be also discussed, along with the possible implications of the current COVID-19 pandemic in the context of CF inflammation and its treatment.

## 2. Anti-Inflammatory Therapy in Cystic Fibrosis 

### Traditional Anti-Inflammatory Treatments

Traditional therapeutic strategies, as well as some more recently studied anti-inflammatory drugs, have shown several drawbacks and limited clinical benefits [13,14]. During the past few years, an increased understanding of the cellular and molecular mechanisms involved in airway infection and inflammation in CF has led to the development of novel therapeutic approaches, many of which are currently under evaluation. The ideal anti-inflammatory treatment in CF should be able to attenuate, but not completely inhibit, the inflammatory process, in order to prevent the risk of infection-related adverse events. Unfortunately, this is not an easy task to achieve.

Corticosteroids are anti-inflammatory molecules with a broad spectrum of activities, targeting many proteins and mediators involved in inflammation [15,16]. Studies of the effects of systemic corticosteroids (prednisone) in CF patients showed that, despite decreasing lung inflammation and preventing lung function decline, their chronic use is limited by important adverse events related to abnormalities in glucose metabolism, growth defects in children, osteoporosis, and cataracts. Of note, the risk of developing these adverse effects was greater compared to the improvement of lung function or reduction of pulmonary exacerbations [17]. Nonetheless, oral corticosteroids are promptly used to decrease CF lung inflammation during an exacerbation. Some trials have also been performed with inhaled glucocorticoids in CF patients. Although they have a better safety profile than oral corticosteroids, current evidence is insufficient to prove their efficacy [18,19]. Thus, the use of inhaled corticosteroids may be appropriate in CF patients with signs and symptoms of asthma or allergic bronchopulmonary aspergillosis, while they are not recommended in patients without these comorbidities, and more studies are needed to better define their use in the CF population.

The only anti-inflammatory treatment currently recommended to CF patients is ibuprofen [20]. This nonsteroidal anti-inflammatory drug prevents the synthesis of prostaglandins by inhibiting the cyclooxygenase enzyme pathway and, at higher doses, the synthesis of leukotrienes through the inhibition of the lipoxygenase pathway [13]. The anti-inflammatory effect of ibuprofen in CF is mainly linked to its ability to decrease neutrophil chemotaxis at inflammatory sites, but the exact mechanism behind this action remains unknown. Additional mechanisms may contribute to the anti-inflammatory activity observed at high doses of the drug, as reported in preclinical studies. It has been shown that ibuprofen can inhibit the activation of NF-κB and AP-1, two important proinflammatory transcription factors [21]; activate peroxisome proliferator-activated receptor (PPAR) alpha and gamma in CF airway epithelial cell lines [22,23]; and modulate inflammatory signaling through the elongation of intracellular microtubules and restoration of microtubule-related transport [24]. On the other hand, at low doses, ibuprofen might increase neutrophil recruitment and have proinflammatory effects [14].

Of note, several studies have highlighted the ability of ibuprofen to interact with the CFTR protein, although results are discordant. Devor and Schultz reported an inhibitory effect of ibuprofen on CFTR function in human colonic and airway epithelia [25], whereas Carlile and colleagues suggested that ibuprofen can activate the channel [26]. Finally, Li and colleagues observed that ibuprofen can either inhibit or stimulate CFTR activity depending on the intracellular levels of cAMP, with inhibition occurring at endogenous levels of the second messenger [27]. In addition, a clinically relevant drug–drug interaction between ibuprofen and lumacaftor/ivacaftor has been recently observed in pediatric CF patients. In particular, it has been shown that lumacaftor may cause subtherapeutic ibuprofen plasma concentrations as a consequence of CYP enzyme induction and increased metabolism of the drug [28]. Thus, further studies are needed to better define the effect of ibuprofen and other nonsteroidal anti-inflammatory drugs on CFTR activity, as well as the potential interactions between these drugs and the novel therapeutic strategies designed to increase either CFTR expression and/or function.

Several trials showing beneficial effects of ibuprofen as an anti-inflammatory treatment in CF patients have been carried out. In 1995, Konstan and colleagues reported that CF patients with mild disease treated with high doses of ibuprofen for a 4-year period had a significantly slower decline in lung function (measured as forced expiratory volume in 1 s—FEV1) and a better body weight as compared to subjects treated with placebo [29]. Subsequently, Lands et al. carried out a 2-year trial with high doses of the drug in 142 CF children 6 to18 years of age with a FEV1 > 60%. Patients treated with ibuprofen showed a reduced rate of forced vital capacity (FVC) but not an FEV1 decline, and a shorter duration of hospitalization, thus supporting that the drug is capable of slowing the progression of lung disease [30]. Interestingly, in 2018, Konstan et al. studied a cohort of CF children followed in the Epidemiologic Study of Cystic Fibrosis and the Cystic Fibrosis Foundation Patient Registry (CFFPR) and showed that high-dose ibuprofen decreases the rate of lung-function decline and improves long-term survival. However, the survival advantage was only observed in children with a baseline FEV1 in a range between 60 and 100% predicted, emphasizing the relevance of selecting the right subgroup of patients for ibuprofen treatment [31]. The ability of high-dose ibuprofen to slow the progression of lung disease in CF patients has recently been confirmed by Lands and colleagues [32]. On the basis of these results, the Cystic Fibrosis Foundation recommends the long-term use of high-dose ibuprofen in children aged 6–17 years with an FEV1 > 60% predicted. However, it is important to monitor drug serum concentrations in order to keep levels in the range of 50 to 100 mg/mL, and to closely monitor patients for early detection of adverse events. Due to the need for close monitoring of drug serum levels, as well as to the possibility of significant, although rare, adverse events, treatment with high-dose ibuprofen is not widely used in clinical practice [17].

## 3. Novel Therapeutic Strategies

### 3.1. Azithromycin

After the first report by a Japanese group of the relevant effect of macrolides on panbronchiolitis, a chronic inflammatory process mainly localized in respiratory bronchioles, a wide number of studies focused on the anti-inflammatory and immunomodulatory effects of this class of antibiotics. Among the different macrolides, azithromycin demonstrated the most interesting effects, and several clinical studies showed the efficacy of this drug in CF patients. This resulted in a widespread use of the drug as chronic treatment intended to modulate the inflammatory process in these patients. In the US, azithromycin is prescribed as maintenance therapy to a considerable number of CF patients (nearly 70%) with chronic airway infection by *P. aeruginosa*; fewer but still a significant number of patients without chronic infection are also treated with this drug [33].

The mechanism of the immunomodulatory action of azithromycin is still not fully understood. In vitro studies have shown that the drug is able to reduce neutrophil chemotaxis at the inflammatory sites, as well as the production of IL-8 and GM-CSF by bronchial epithelial cells [34,35]. A recent report also showed that it induces a modification of the M2 phenotype of macrophages and inhibits the activation of NF-κB [36]. Furthermore, clinical studies showed that treatment with azithromycin induced a decrease in IL-8 production and the release of neutrophil elastase (NE) in CF patients infected with *P. aeruginosa*, while in *P. aeruginosa*-negative patients, a reduction of serum amyloid A, calprotectin, C-reactive protein, and absolute neutrophil count was observed [37,38]. Interestingly, Saint-Criq et al. also reported a positive effect of azithromycin on the restoration of Cl- efflux in CF [39].

Several studies have been performed with the chronic use of oral azithromycin over a 6-month period, while only few studies have evaluated the long-term impact of this treatment on CF patients. A Cochrane meta-analysis reviewing the most relevant randomized controlled trials carried out with azithromycin administered for 6 months in both adults and children (>6 years old), with or without chronic *P. aeruginosa* infection, showed a significant improvement of FEV1, associated with a decreased number of pulmonary exacerbations, a reduction of the use of oral antibiotics, and a weight gain in treated patients. Nevertheless, evidence from this study was insufficient to recommend or preclude azithromycin use in patients without chronic *P. aeruginosa* infection. The treatment appeared to be safe, but emergence of macrolide resistance was a concern [40]. Subsequently, a study by Nichols and colleagues suggested a negative interaction between azithromycin and inhaled tobramycin, two of the most commonly prescribed drugs in CF patients. In particular, these authors reported that azithromycin may induce the expression of *Pseudomonas* efflux pumps that reduce the intracellular concentration of tobramycin [41]. In a more recent study, Nichols and collaborators analyzed the relationship between the chronic use of azithromycin and the concomitant use of inhaled tobramycin on data obtained from the CFFPR. In this study, patients were divided into two cohorts: *P. aeruginosa*-positive and -negative patients treated for at least three years. A reduced FEV1 decline was observed only in *P. aeruginosa*-positive patients. In a subset of patients treated with both azithromycin and tobramycin, the limitation of FEV1 decline was not observed, supporting the previous hypothesis of a negative interaction between the two drugs.

At present, the Cystic Fibrosis Foundation recommends the use of chronic oral azithromycin treatment in patients > 6 years old with chronic *P. aeruginosa* infection. The drug use could also be taken into consideration for *P. aeruginosa*-negative patients. All subjects must be screened for nontuberculous mycobacteria before starting the treatment, and then every 6–12 months.

### 3.2. Compounds Affecting Eicosanoid Pathway

Leukotriene B4 (LTB4) is a potent neutrophil chemoattractant that is produced by bronchial epithelial cells and activated inflammatory cells, and is involved in the amplification of the inflammatory response in CF. The amount of this mediator is increased in CF airways and plays a crucial role in neutrophil recruitment. A large Phase II/III clinical trial with the LTB4 receptor antagonist BIIL 284 BS in adults and children with CF was stopped early because adults receiving this treatment had increased frequency of pulmonary exacerbations and serious adverse events. This likely occurred because of the potent anti-inflammatory effect of the compound, and it emphasized the importance of not switching off completely the inflammatory response in CF [42].

More recently, acebilustat (CTX-4430), a novel inhibitor and modulator of leukotriene A4 (LTA4) hydrolase, has been evaluated in CF. LTA4 hydrolase is an enzyme that generates LTB4 from leukotriene A4. By inhibiting LTA4 hydrolase, acebilustat decreases neutrophil influx at inflammatory sites and the release of NE. Moreover, it has been shown that LTA4 hydrolase inhibition leads to the production of the proresolving lipid mediator lipoxin A4 (LXA4). In 2017, Elborn and colleagues performed a pharmacokinetic and pharmacodynamic study of this new drug in CF patients, and they did not observe any difference compared to healthy volunteers, since LTB4 production was inhibited either in blood or sputum of both study populations. Subsequently, the same authors performed a Phase I study with two doses of acebilustat (50 mg or 100 mg) for 15 days in CF patients with mild or moderate disease. Compared to placebo, acebilustat induced a significant reduction of neutrophil counts and elastase in the sputum, but no changes were observed in pulmonary function. The drug was safe and well tolerated. A Phase II, multicenter, randomized, placebo-controlled study was recently carried out in adults with CF in order to evaluate the efficacy, safety, and tolerability of acebilustat administered orally once daily for 48 weeks (NCT02443688). The results of this study are expected soon.

### 3.3. Cannabinoid-Derived Compounds 

The resolution of chronic inflammation is a major unmet medical need in CF. The synthetic cannabinoid-derived lenabasum could provide a safe and effective drug for this purpose. Lenabasum is a synthetic and selective agonist of cannabinoid receptor type 2 (CB2) on immune cells. This drug is able to trigger the resolution phase of the innate immune response, has potent anti-inflammatory effects, and modulates the fibrotic response without inducing any adverse effects on the central nervous system [43].

In preclinical models, at a dose of 5 mg lenabasum was shown to inhibit the production of LTB4, neutrophil infiltration, and the activity of antiphagocytic prostanoids (PGE2, TxB2, and PGF2α). At higher doses, it was also able to induce the production of proresolving lipid mediators, such as LXA4 and LXB4, as well as resolvin D1 and D3 [44]. More recently, Tarique et al. reported that lenabasum was able to induce CF macrophage polarization toward the proinflammatory M1 phenotype, improving cell phagocytic activity, and to inhibit the production of proinflammatory cytokines such as IL-8 and TNF-alpha [45].

In Phase I and II clinical trials, the compound demonstrated a favorable safety and tolerability profile. Moreover, in an early, small Phase II trial, lenabasum was shown to reduce multiple markers of inflammation in CF patients. Recently, Chmiel and colleagues carried out a phase II, randomized, placebo-controlled study in stable CF patients with FEV1 > 40%. Lenabasum was administered at a dose of 1 or 5 mg for 4 weeks, and afterward treated patients received 20 mg once or twice daily for another 8 weeks. Lenabasum pharmacokinetic was similar in CF patients and controls. Furthermore, the study showed that the drug was able to reduce the risk of pulmonary exacerbations associated with a reduction of inflammatory markers (elastase activity, IL-8, and IgG) [46]. However, a larger Phase IIb trial did not meet its primary objective of decreasing pulmonary exacerbations in people with CF [47].

Further trials are ongoing and are needed to better define the use of this drug in CF patients.

### 3.4. R-Roscovitine 

R-roscovitine is a cyclin-dependent kinase (CDK) inhibitor competing with the binding of ATP to the enzyme that has been used as a pharmacological tool to investigate cell functions, such as cell-cycle control and apoptosis. Roscovitine has also been evaluated as a drug candidate in several diseases, such as neuroblastoma, viral infections, rheumatoid arthritis, and others [48].

Although the mechanism of action of roscovitine is not fully understood, this molecule has several properties that suggest a potential therapeutic benefit in CF. It favors the trafficking of the mutant F508del-CFTR to the plasma membrane, partially protecting the protein from proteolytic degradation [49]. Furthermore, it promotes the resolution of inflammation by triggering neutrophil apoptosis, through the restoration of the Th17/Th2 balance and the reduced production of some cytokines [50,51,52]. Moreover, it rescues the acidification of phagolysosomes in CF alveolar macrophages, improving their bactericidal activity [53]. A Phase II, double-blind, placebo-controlled trial to evaluate the safety and efficacy of roscovitine in CF patients with at least one copy of F508del, chronically infected with *P. aeruginosa*, is ongoing in France [11].

### 3.5. Thymosin Alpha-1

Thymosin alpha 1 (Tα1) is a human, naturally occurring polypeptide that is used worldwide as an immunomodulator in several diseases, such as viral infections, immunodeficiencies, malignancies, and HIV/AIDS.

In 2017, Romani and colleagues evaluated the potential use of Tα1 in CF by assessing its effect on CF airway epithelial cell lines (CFBE41o- cells) in vitro, and in homozygous F508del-CFTR C57BL/6 mice in vivo. Tα1 was shown to inhibit the inflammatory response both in cell lines and in CF mice. In vitro, Tα1 rescued the indoleamine-pyrrole 2,3-dioxygenase expression in epithelial cells. Both in vivo and in vitro, this drug decreased the production of proinflammatory cytokines, such as TNF-α, IL-1β, and IL-17A, while increasing IL-10. Moreover, in naïve and infected CF mice, it inhibited neutrophilic infiltration and the development of CF-like disease in the lungs. Interestingly, the study also showed that Tα1 was capable of increasing CFTR maturation, stability, and activity [54,55]. Nevertheless, this latter effect was not confirmed by subsequent studies [56,57]. More recently, the group of Romani and colleagues suggested that Tα1 can also have beneficial effects in CF extrapulmonary pathology. Specifically, they showed that the molecule was able to restore barrier integrity and immune homeostasis in the inflamed gut of CF mice, as well as in mice with the metabolic syndrome [58]. However, further studies are needed to better define the efficacy and the potential use of this molecule in CF patients.

## 4. Alternative Strategies 

### 4.1. Inhibitors of Neutrophil Elastase

CF airway inflammation is characterized by a marked and persistent neutrophil recruitment associated with the release of large amounts of NE into the airways; this mediator plays a crucial role in tissue destruction and remodeling, and in the progressive impairment of lung function. Two different pharmacological approaches have been studied to inhibit protease activity in CF airways: either to increase anti-protease levels, or to inhibit protease expression. Several studies have searched for effective and safe protease inhibitors and assessed their potential use in CF [59]. The main problem was to reach effective concentrations of these inhibitors in the airways that were capable of binding all free NE present at this site.

Human alpha-1 antitrypsin (AAT) is still the most studied drug by far, and several clinical trials were already carried out with this drug. A Phase IIa, randomized, placebo-controlled, multicenter clinical trial with a new formulation of inhaled alpha-1 proteinase inhibitor (Alpha-1 HC) administered for 3 weeks was carried out by Gaggar and collaborators in 30 adult CF patients. There was a dose-dependent increase of sputum levels of alpha-1 proteinase inhibitor at the end of the study, and the drug was safe and well tolerated. However, no effect on lung function was observed, although the study was not powered to assess these effects. Furthermore, the effects were transient and difficult to predict due to proteases variability in the lungs of CF patients [60]. Unfortunately, due to logistical and financial problems, the study of this drug has been interrupted.

Another serine protease inhibitor is the secretory leukoprotease inhibitor (SLPI), an antiprotease produced by airway epithelial cells and neutrophils that contributes to maintain the protease/anti-protease balance in the airways, and to prevent protease-mediated tissue destruction. Different approaches have been proposed to increase the antiprotease activity by nebulizing SLPI, but the efficacy of this compound is still being evaluated [61,62].

Recently, novel protease inhibitors of promising interest in the CF context are in development, such as POL6014, DX-890, AZD9668, and Grifols T6006-201. POL6014 is a highly selective, potent, and reversible NE inhibitor that is administered by inhalation through the Pari eFlow system. Preclinical data have provided evidence that this molecule selectively inhibits NE in enzymatic assays and reduces neutrophils as well as inflammatory markers in animal models of neutrophilic inflammation. The safety and pharmacokinetic profile of POL6014 was assessed in healthy volunteers and in CF patients by Barth and colleagues [63]. The drug was able to reach high concentrations in the lungs and to decrease and inhibit NE in broncho-alveolar lavage and sputum of CF patients. Further studies are needed to evaluate if there are clinically relevant effects associated with the reduction of NE in CF.

Very recently, novel approaches using AAT gene therapy are emerging. Recent data have provided encouraging results with the inhibition of miRNAs that target the mRNA of SERPINA1, encoding AAT, by using antagomirs or target-site blockers [64]. Another possible approach is to directly activate SERPINA1 by using viral vectors like retroviruses or adenoviruses, but numerous side effects have been observed [65].

CF lung damage can also be driven by MMPs, a group of enzymes that regulate inflammatory and repair processes. They have been shown to be upregulated in the sputum of CF patients and to be related to lung damage [66,67]. Activation of metalloproteases can be induced by proteases and is modulated by tissue inhibitors of metalloproteases. Several recent clinical studies of CF focused on modulation of MMP activity to improve disease outcome. A Phase II study with Andecaliximab/GS-5745 is in progress in adult patients with CF, and this could represent a novel approach to control tissue damage [19].

### 4.2. Compounds Affecting Lipid Metabolism

Several studies have suggested an alteration of lipid metabolism in CF. In particular, some studies have reported that ceramide levels are decreased in CF, contributing to the persistence of bacterial infection and the enhanced inflammatory response in the airways of CF patients [68,69]. However, the role of ceramide in CF is still controversial; some authors have demonstrated that ceramide is increased in CF epithelial cells, while others have reported that its concentration is reduced.

Fenretidine (LAU-7b) is an oral synthetic retinoid administered once daily that increases docosahexaenoic acid, and consequently ceramide concentration, thus decreasing the inflammatory response. A Phase II clinical study to test the effectiveness and safety of LAU-7b in CF patients is ongoing [32]. Further clinical studies are needed to better understand the potential of this drug as a novel anti-inflammatory treatment in CF patients.

### 4.3. Compounds Targeting Arginine Production and Nitric Oxide

An anti-inflammatory drug candidate that is currently in the CF drug pipeline is CB-280, an oral drug designed to increase the amount of arginine in the lungs to augment the production of nitric oxide, which could help in fighting lung infections. A Phase 1 study to test the safety and tolerability of CB-280 in adults with CF and chronic *Pseudomonas aeruginosa* infection is currently underway [47].

## 5. New Perspectives 

### 5.1. Stem Cells

During the past few years, there has been a growing interest in the potential use of stem cells in different pathological conditions, including CF. These cells retain the ability to differentiate into a variety of cell lineages and to contribute to the repair and regeneration of injured tissues. In particular, mesenchymal stem cells (MSCs) are multipotent, nonhematopoietic stem cells that are present in many tissues, including the lungs. They have the ability to home to sites of tissue injury and to help repair the injured area by differentiating into resident cells and releasing extracellular vesicles (EVs) that promote cell communication. At the site of injury, MSCs contribute to the modulation of the local microenvironment through the release of angiogenic, anti-apoptotic, and inflammatory modulators. There is growing evidence that MSC and EVs could be immunologically impaired in CF, contributing to the maintenance of pulmonary inflammation. Thus, the potential use of human MSCs as a cell-based therapy in CF is of particular interest [70].

In a study carried out in CFTR-deficient mice with chronic *P. aeruginosa* infection, Bonfield and colleagues demonstrated that human MSCs were able to decrease levels of proinflammatory cytokines as well as weight loss, improving lung pathology associated with chronic infection and clinical score [71]. Subsequently, Sutton and colleagues showed that MSCs were able to enhance the production of anti-inflammatory cytokines, such as IL-6 and CCL2, while decreasing the release of the proinflammatory IL-8. They also reported that healthy MSCs were able to restore the expression of the anti-inflammatory transcriptional regulator PPARγ in CFTR-deficient macrophages [72]. In a subsequent study, Zulueta et al. confirmed that MSCs-derived EVs were capable of partially restoring PPARγ signaling in CF bronchial epithelial cell lines, downregulating the transcription and expression of some pro-inflammatory cytokines (IL-1β, IL-8, IL-6), and partially impairing the nuclear translocation of NF-κB [73]. Furthermore, recent data support the concept that MSCs also exert antimicrobial effects through direct and indirect mechanisms [74]. Overall, these data suggest that human MSCs might represent a potential future anti-inflammatory therapy in CF patients. However, although huge progress has been made in the study of MSCs and MSCs-derived EVs, many challenges remain open. Two Phase I clinical trials to assess the safety and tolerability of allogenic MSCs in adults with CF are ongoing.

### 5.2. Anticytokines 

As mentioned above, the lung environment of CF patients is characterized by high levels of proinflammatory cytokines, such as IL-8, IL-6, and TNF-alpha, and decreased levels of anti-inflammatory mediators, such as IL-10 [75], associated with a marked and persistent neutrophil recruitment into the airways. Thus, the possibility of using cytokine modulators to inhibit the exaggerated inflammation in CF represents a potential rational approach.

At present, there is evidence from preclinical data that antibodies directed against intracellular adhesion molecules and IL-8 might be promising tools [19]. SB-656933, an oral CXCR2 antagonist, has been tested in CF patients, and has demonstrated a good safety profile as well as the capability of modulating airway inflammation with an improvement of sputum inflammatory biomarkers [76]. Recently, Balazs et al. [77] suggested a role for the IL-1 signaling pathway in the pathogenesis of sterile neutrophilic inflammation and mucus hypersecretion in CF, and highlighted the potential role of Anakinra, an IL-1 antagonist, as an anti-inflammatory treatment in CF. Several other cytokine modulators have been studied in vitro for their potential anti-inflammatory activity in CF; some of these molecules are promising, and in vivo studies are needed to assess their potential use in CF patients.

The possibility of targeting miRNA involved in CF lung disease to modulate the inflammatory process or the activity of transcription factors, such as NF-κB, are novel and interesting approaches that also have been pursued more recently [78].

Finally, other interesting approaches to dampen CF airway inflammation have been explored in animal models, such as the inhibition of phosphoinositide 3-kinase γ (PI3Kγ), an enzyme that plays a pivotal role in leukocyte recruitment and activation. Galluzzo and collaborators showed that genetic deletion and pharmacological inhibition of PI3Kγ were able to decrease neutrophilic airway inflammation and structural lung damage in a mouse model of CF lung disease, suggesting that this enzyme may thus represent a potential target for anti-inflammatory treatment in CF [79]. 

Overall, further studies are needed to better define the role of all these novel approaches in CF, as the risks of side effects remain high [19].

## 6. Anti-Inflammatory Effects of CFTR Modulators

### 6.1. Approved CFTR Modulators

Since the late 1990s, major efforts have been made to discover small molecules directed against either the absent or dysfunctional chloride channel in epithelial membranes, leading to the introduction of CFTR modulators, a new category of therapeutic agents designed to target the molecular defects of CF. Depending on the molecular mechanisms through which they enhance or restore the function of the defective CFTR, modulators can be classified into five main subgroups: correctors, potentiators, read-through agents, stabilizers, and amplifiers [80]. Correctors allow the proper maturation and delivery of the channel to the plasma membrane, thus repairing the trafficking/maturation defects of Class II mutants, including the most prevalent F508del-CFTR, while potentiators increase the ion flow through appropriately located, apical CFTR, targeting Class III genetic defects [81,82]. Moreover, read-through agents enable the bypass of premature stop codons caused by Class I CFTR mutations, while the goal of stabilizers and amplifiers is to enhance the stability of the membrane-bound CFTR and its mRNA, respectively [83,84].

To date, four CFTR modulators have been approved for the treatment of CF patients carrying specific CFTR mutations, and the field is in constant expansion due to the promising results obtained with the first molecular drugs. Of note, ivacaftor (formerly VX-770, marketed as Kalydeco^®^), the first CFTR-modulating drug to receive U.S. Food and Drug Administration (FDA) approval in 2012, drastically changed the treatment of nearly 10% of CF patients. It was demonstrated to improve lung function, sweat chloride, and the overall quality of life in subjects carrying Class III gating mutations [85,86]. Unfortunately, because ivacaftor serves as a CFTR potentiator, the great majority of CF subjects carrying the Class II F508del mutation could not benefit from the treatment [87]. A slight improvement in lung function was observed in these patients when ivacaftor was coadministered with lumacaftor (formerly VX-809) [88], a first-generation CFTR corrector that previously showed disappointing results in a Phase IIa clinical study on F508del-homozygous patients [89]. These findings led to the introduction of the combination therapy, ivacaftor + lumacaftor, marketed as Orkambi^®^, in 2015. Another CFTR corrector, tezacaftor (VX-661), with improved pharmacokinetic properties and fewer collateral effects (such as lower cytochrome P450 3A activation) than lumacaftor when used in combination with ivacaftor, was subsequently developed by Vertex Pharmaceuticals [90,91]. Accordingly, the tezacaftor/ivacaftor dual therapy (Symdeko^®^) was approved in 2018 for the treatment of Orkambi-intolerant CF patients with homozygosity for the F508del mutation or with a single copy of one of 26 specified mutations [92]. 

**Figure 1:**
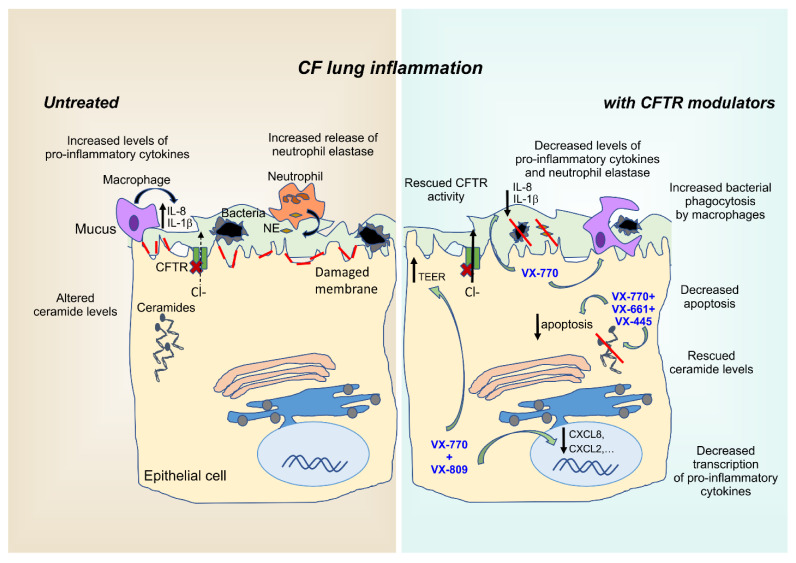
Anti-inflammatory properties of CFTR modulators. CF airways are a highly inflammatory environment characterized by infiltration of inflammatory cells, primarily neutrophils, that fail to effectively clear pathogens entrapped in the abnormally thick and sticky mucus layer. Consequently, failed resolution of this indiscriminate and sterile immune response, further fueled by the abnormal intracellular ceramide levels of airway epithelial cells, disrupts the physiological lung barrier, resulting in reduced TEER values. Besides restoring CFTR activity, CFTR modulators exert various anti-inflammatory effects by targeting intracellular processes, such as ceramide accumulation, cytokine transcription, membrane integrity, and macrophage-mediated phagocytosis of bacteria. Red lines represent the damaged plasma membrane. TEER: transepithelial electrical resistance; NE: neutrophil elastase; VX-770: ivacaftor; VX-809: lumacaftor; VX-661: tezacaftor; VX-445: elexacaftor.

Nevertheless, all these corrector–potentiator combinations demonstrated only modest efficacy in F508del-homozygous patients. These observations highlighted the need for additional high-throughput screenings (HTSs) aimed at identifying next-generation correctors with different mechanisms of action that could be combined in triple combinations to ensure synergistic effects. Among four novel small molecules targeting the intracellular processing and trafficking of F508del-CFTR, VX-445 (elexacaftor) emerged as the most promising candidate. VX-445 showed a good safety profile for long-term use, which was confirmed in Phase II and III trials of the triple therapy combination including, in addition to VX-445, tezacaftor and ivacaftor. This cocktail was recently approved as Trikafta^®^ [93,94] and is expected to become a game-changer in the treatment of 90% of CF patients (for extensive review on CFTR modulators, refer to [95]). In addition to ensuring significant improvements in parameters of lung function, such as FEV1, and a decrease in pulmonary exacerbations, CFTR modulators also showed the potential of reducing airway inflammation (Figure 1), as we will describe in detail in the following paragraphs.

### 6.2. Ivacaftor Monotherapy

Earlier studies found that inflammatory markers did not decrease in sputum samples of patients carrying the Class III mutation G551D that were undergoing ivacaftor therapy, although the relative abundance of several common pathogens in CF displayed a clear downward trend [96,97]. In contrast, recent evidence highlighted that ivacaftor not only lowered the sputum concentrations of *P. aeruginosa* throughout the first year of treatment, but also the levels of NE, IL-8, and IL-1β in patients carrying the same G551D mutation [98]. However, the *P. aeruginosa* infection was not eradicated in any of the subjects studied, indicating that additional antibiotic treatments are still required in CF therapeutic regimens. In terms of overall bacterial burden and interaction with antibiotics, the potentiator was found to inhibit the growth of respiratory isolates of *Streptococcus aureus* and *Streptococcus pneumoniae*, and to preserve antibiotic susceptibility in vitro [99,100], allowing its coadministration with common antimicrobial agents. In particular, recent analyses showed that ivacaftor enhances the antibacterial activity of ciprofloxacin, a broad-spectrum antibiotic of the fluoroquinolone class often included in CF therapy [101]. Moreover, physiological cytosolic ion levels (chloride, sodium, and magnesium) and activation of Rab27a, both required for effective degranulation and bacterial killing of neutrophils, were restored upon ivacaftor treatment of G551D patients [102].

Overall, these data highlight the potential anti-inflammatory activity of ivacaftor, suggesting additional clinical benefits of therapy with CFTR modulators. Despite these optimistic results, patients treated with ivacaftor still rely on traditional antimicrobial and anti-inflammatory treatments in order to avoid pulmonary exacerbations and dampen airway inflammation [103,104].

### 6.3. Combination Therapies: Orkambi^®^ and Symdeko^®^

After the initial approval of the dual therapy Orkambi^®^, interesting data showed that the functional rescue of F508del-CFTR also significantly reduces the mRNA levels of CXCL8, CXCL1, and CXCL2 in response to *P. aeruginosa* exposure, highlighting the potential anti-inflammatory properties of the corrector/potentiator combination [105]. Moreover, two Phase III, randomized, double-blind, placebo-controlled studies performed on F508del-homozygous patients showed that the rate of pulmonary exacerbations was decreased in the lumacaftor–ivacaftor groups, requiring fewer hospitalizations or intravenous antibiotic treatments [106]. However, macrophage phagocytosis, which is markedly impaired in CF lungs and partly responsible for the abnormal hyperinflammatory environment [107,108], was restored in patients taking ivacaftor, but not lumacaftor/ivacaftor [109]. These findings suggest that a negative drug combination may occur between the potentiator and the first-generation corrector in terms of stimulation of bacterial phagocytosis by CF macrophages. This hypothesis was confirmed by further analyses that detected a decreased ability of lumacaftor to induce killing of *P. aeruginosa* upon coadministration with ivacaftor [110]. Furthermore, recent whole-blood transcriptomic analyses revealed that F508del-homozygous patients displayed a significant and persistent overexpression of an array of inflammatory genes compared to non-CF controls, which remained unchanged upon therapy with lumacaftor/ivacaftor [111]. In contrast, a study by Adam and collaborators demonstrated that primary airway epithelial cell cultures from patients with Class II mutations exhibit a modest but relevant improvement in transepithelial resistance and cellular repair after treatment with Orkambi^®^, despite the presence of *P. aeruginosa* exoproducts. Therefore, the first FDA-approved combination of CFTR modulators favors the integrity of the airway epithelium in vitro, a key factor for the maintenance of lung homeostasis and the absence of abnormal inflammatory response.

Moreover, a recent study demonstrated that CF peripheral blood mononuclear cells (PBMCs) pretreated with ivacaftor alone, or combined with lumacaftor, exhibited reduced levels of ROS upon challenge with *Aspergillus fumigatus*. These findings, suggesting that these CFTR modulators might have additional immunomodulatory benefits to prevent or treat *Aspergillus*-induced inflammation [112], are particularly relevant considering that *A. fumigatus* colonization is frequent in CF and worsens pulmonary exacerbations [113]. However, whether lumacaftor/ivacaftor cotreatment may protect against acute exacerbations and airway inflammation in CF patients is still controversial, and further research is needed to clarify this point. On the other hand, a study by Gentzsch and colleagues highlighted that the infectious/inflammatory milieu of CF airways can enhance VX-809-mediated rescue of F508del-CFTR, demonstrating the existence of a dose–response relationship between airway inflammation and efficacy of CFTR modulators in vitro [114]. This evidence further supports the notion that a complete suppression of the inflammatory response in CF airways may not be beneficial.

Because inflammation is a complex process that relies on the cooperation of different cell subtypes, to evaluate the effects of combination therapies on the distinct immune cell subpopulations, and on their secretome, may lead to a better understanding of the anti-inflammatory potential of such drugs. Of note, Pohl and collaborators [115] investigated in different leukocytes the levels of the CXCR2 receptor, which is bound by the neutrophil chemoattractant IL-8, an inflammatory cytokine with severe implications in the pathophysiology of CF [116]. Interestingly, CXCR2 was found to be increased on the surface of monocytes from F508del-homozygous patients who discontinued the use of Orkambi^®^ due to adverse effects, compared to subjects still on lumacaftor/ivacaftor treatment. Although the mechanisms and biological relevance of elevated CXCR2 levels in these patients still lack a clear explanation, this chemokine receptor may promote leukocyte migration and subsequent lung colonization by immune cells, ultimately leading to airway hyperinflammation. Hence, Orkambi^®^ might potentially prevent these events by avoiding an exaggerated expression of CXCR2.

Concerning the concentration of interleukins, a recent study demonstrated that serum levels of IL-18 and TNF were significantly reduced upon treatment with both Orkambi^®^ and Symdeko^®^ in patients with F508del in homozygosity, but only the tezacaftor/lumacaftor combination decreased serum IL-1β [117]. The different behavior of Orkambi^®^ and Symdeko^®^ unveils a novel anti-inflammatory property exclusive to coadministration of tezacaftor, which may thus exert a wide range of therapeutic effects against lung infection and inflammation, both associated with IL-1β. Furthermore, very recent results from Shrestha and colleagues identified a synergistic effect between R-roscovitine, a synthetic tri-substituted purine that has shown multiple benefits in CF [48,49], and Symdeko^®^ in the killing of *Burkholderia cenocepacia* by CF macrophages [118], thus disclosing new encouraging perspectives on the use of the tezacaftor/ivacaftor combination to dampen CF inflammation.

Contrasting evidence emerged from enriched gene ontology analyses performed on CF lung epithelial cells treated with different CFTR modulators. In particular, lumacaftor-induced differential expression included a small set of downregulated genes involved in immune, inflammatory, and interferon signaling processes, while tezacaftor did not alter the transcript levels of enough genes to exhibit enriched ontologies [119]. Considering that Symdeko^®^ was approved only in 2018, a deeper investigation of the anti-inflammatory properties of tezacaftor and its combination therapy with ivacaftor is still ongoing.

### 6.4. Triple-Therapy: Trikafta^®^

The introduction of the elexacaftor/tezacaftor/ivacaftor combination in therapeutic regimens of CF patients 12 years of age and older with at least 1 F508del mutation, regardless of their second mutation type [120], increased the eligibility for CFTR-modulating treatments to ~90% of CF subjects [121]. Importantly, the addition of the next-generation corrector elexacaftor lowered the annual rate of pulmonary exacerbations by 63% in comparison to F508del homozygotes receiving Symdeko^®^, which showed a reduction of 35% versus placebo [122]. Notably, an in vitro study by Liessi and collaborators [123] highlighted that the triple combination is the only treatment able to concomitantly and significantly lower the levels of 6 different ceramides in CFBE41o- cells, which are known to trigger inflammation and death by accumulating in CF epithelial cells [124]. Moreover, since these sphingolipids can act as second messengers in the stimulation of apoptosis [125,126], Trikafta^®^ may decrease the susceptibility of epithelial cells to death in response to severe proapoptotic stimuli, such as in vitro application of etoposide. This effect of the latest approved combination therapy could open interesting new perspectives in the treatment of CF patients. In contrast, the same study revealed a reduced consumption by Trikafta^®^-treated cells of lysophosphatidylcholines, chemotactic lipids that promote macrophage infiltration [127]. As a consequence, the high extracellular concentrations of these chemoattractants could potentially favor the recruitment of a higher number of immune cells to Trikafta^®^-treated lungs, further fueling airway inflammation. Therefore, the preliminary contrasting results on the anti-inflammatory properties of the elexacaftor/tezacaftor/ivacaftor combination suggest that further in-depth analyses are required to clarify the role of Trikafta^®^ in modulating CF hyperinflammation.

### 6.5. Iminosugars, TMA Analogs, and Proteostasis Regulators

In addition to the approved treatments described above, there are various molecules that have proven beneficial in both correcting the basic defect of CF and reducing airway inflammation in cellular models and patients. In particular, it is known that iminosugars, which are natural or synthetic compounds that resemble carbohydrate substrates or saccharide hydrolysis transition states [128,129], display an interesting pharmacological potential as CFTR correctors. The N-alkylated iminosugar miglustat (NBDNJ), an inhibitor of the glucosylceramide synthase, was found to affect sphingolipid metabolism in CF bronchial epithelial cells, thus limiting the inflammatory response elicited by *P. aeruginosa* [130]. At the same time, this molecule was shown to inhibit the α-glucosidase of the ER and restore F508del-CFTR trafficking [131]. Unfortunately, Phase II clinical trials did not confirm the in vitro efficacy of NBDNJ on chloride transport in CF patients [132]. Notably, a specific analogue of adamantanemethyloxypentyl-1-deoxynojirimycin (AMP-DNM), a potent inhibitor of the non-lysosomal β-glucosidase 2 enzyme, involved in sphingolipid metabolism and *P. aeruginosa*-induced inflammation [133], proved to effectively correct F508del-CFTR in CF-KM4 cells [134]. Therefore, this iminosugar exhibits relevant anti-inflammatory and corrector properties, and represents an intriguing potential therapeutic agent for CF treatment.

Another interesting molecule is 4,6,4′-trimethylangelicin (TMA), a synthetic derivative of angelicin that has been demonstrated to act as both a corrector and a potentiator of mutant CFTR [135,136], as well as a strong inhibitor of the expression of IL-8 in *P. aeruginosa*-challenged bronchial epithelial cells [135]. Importantly, in vitro screenings of TMA analogues identified five compounds with improved inhibitory activity of IL-8 gene expression, with minimal adverse effects on cell proliferation [137]. Thus, these promising results may translate into in vivo benefits for patients experiencing intolerance to conventional CF drugs.

Finally, proteostasis regulators may also be exploited to achieve anti-inflammatory effects. Proteostasis is dramatically altered in CF cells because of the ability of the CFTR to serve as a hub protein coordinating a proteostatic network, also involving intracellular chaperone machineries [138]. In particular, CFTR dysfunction has been shown to negatively impact autophagy [139], while causing the overactivation of CK2 kinase [140], with a consequent increase in lung inflammation and channel degradation. A relevant study by Tosco and coworkers [141] showed that a combination treatment including the cystinosis drug cysteamine [142] and the flavonoid epigallocatechin gallate, two safe proteostasis regulators, reduced inflammatory cytokines by 30% in nasal cells and sputum of CF patients bearing at least one copy of Class II CFTR mutations, while concomitantly restoring CFTR function. Moreover, recent in vitro analyses on a novel cysteamine–dendrimer formulation (G4-CYS), with a core composed of two cysteamines linked by a disulfide bond, demonstrated that this compound effectively rescues F508del-CFTR by allowing its correct translocation to the plasma membrane and inhibits *P. aeruginosa* bacterial infection by restoring autophagy [143]. Nevertheless, the efficacy of cysteamine is still a matter of debate, and further studies are awaited to clarify this point.

## 7. Potential Impact of COVID-19 on Inflammation in CF Patients

Viral infections represent the causative factor of ~60% of acute pulmonary exacerbations in CF patients [144,145], partly due to the reduced antiviral immunity of lung epithelial cells, which subsequently allow the uncontrolled replication of the infective agent [146,147]. Flight and collaborators [148] performed a large-scale, prospective study to assess the incidence of viral infections in adults with CF, with rhinovirus accounting for ~73% of them, and found a strong association with pulmonary exacerbations, disease progression, and increased morbidity, as previously reported [149,150,151,152]. Moreover, the outburst of the H1N1 influenza pandemic in 2009–2010 determined substantial morbidity in the majority of CF subjects who contracted the infection [153].

The ongoing global health emergency caused by the novel SARS-CoV-2 virus, responsible for coronavirus disease 2019 (COVID-19) [154], has immediately raised the question regarding whether CF patients could be more susceptible to SARS-CoV-2 infections than the general population. Recent evidence indicates that infection with SARS-CoV-2 does not cause worse outcomes in CF patients. In particular, an early multinational report on 40 CF cases highlighted good recovery from SARS-CoV-2 in the heterogeneous 40-subject CF cohort, with a total absence of mortality that may derive from the relatively low incidence of the novel coronavirus amongst the CF population [155]. A subsequent report involving a larger international cohort (181 people with CF) confirmed that SARS-CoV-2 infection for most people with CF may be less severe than originally feared, although it can result in serious consequences for some CF patients (with older age, CF-related diabetes, lower lung function, and recipients of an organ transplant) [156]. Moreover, it has recently emerged that a one-month-old infant with CF and SARS-CoV-2 positivity did not display any COVID-19 symptoms, suggesting that the low pathogenicity observed in healthy children applies to young CF patients as well [157].

Considering that the SARS-CoV-2 infection triggers a cytokine storm, comprehensive of type I and type III interferons [158,159], infected CF patients already disclosing cytokine dysfunction and hyperinflammation were reasonably expected to be at high risk of severe respiratory disease. The mitigating effects observed in CF subjects infected by SARS-CoV-2 have been recently linked to different molecular mechanisms. The virus exploits its spike coat protein to bind the host angiotensin-converting enzyme2 (ACE2), which different lung cells, comprising the airway epithelial subtype, exhibit on their plasma membrane [160]. Subsequent cleavage or “priming” by the serine protease TMPRSS2 allows successful cell entry and host-cell colonization [161]. Importantly, a deep examination of publicly available data by Stanton et al. [162] recently highlighted that transcript levels of ACE2 and TMPRSS2 are elevated and decreased in CF airway epithelial cells, respectively. Taking into account that ACE is responsible for the cleavage of angiotensin I (ANG I) to angiotensin II (ANG II), and that ACE2 processes ANG II to the anti-inflammatory agent angiotensin-1–7, higher concentrations of the SARS-CoV-2-bound enzyme ACE2 indirectly hampers airway inflammation [163,164]. Hence, preliminary speculations suggest that the viral entry via ACE2 may be impaired in CF patients, further protecting them from developing COVID-19. 

Taken together, these preliminary data on the potential impact of SARS-CoV-2 infection in CF patients delineate a more positive picture than anticipated, suggesting that airway hyperinflammation and overall lung susceptibility resulting from the underlying genetic defect of CFTR do not worsen COVID-19 manifestation. Further studies on the impact and outcomes of SARS-CoV-2 infection in CF patients might provide novel and useful information on antiviral defense mechanisms in CF and on the relationship between viral infections and the inflammatory responses in this disease.

## 8. Conclusions

Increasing evidence has accumulated demonstrating that an exaggerated and dysregulated inflammatory response occurs in the CF airways, and plays a crucial role in the irreversible lung damage and the progressive lung-function decline. The inflammatory process in CF begins early in the natural history of the disease and remains persistent and self-perpetuating through life, making it a relevant therapeutic target.

As traditional anti-inflammatory treatments in CF have shown several limitations, the identification of innovative and more specific approaches still represents an area of intense investigation. In the last decade, an increased understanding of the cellular and molecular mechanisms of CF airway inflammation has led to the identification of critical molecular targets, and several ongoing studies that are now in the evaluation phase have shown that new approaches could be very promising in inhibiting the ongoing inflammatory process in CF.

During the past few years, most of the research efforts in the CF field have focused mainly on the discovery and study of CFTR modulators that address the basic defect of CF. Although these drugs have shown some effects on inflammation, these are indirect and variable, and their impact on the inflammatory responses is not clearly defined yet. Furthermore, although CFTR modulators would halt the progression of CF lung disease, their effects on inflammation may be closely tied to when, in the disease process, the drugs will be started, and to the degree of underlying structural lung damage, as well as to the improvement in airway infection. Thus, despite the dramatic impact that CFTR modulator therapy will likely have on the natural history of CF lung disease over the coming years, considering the central role of inflammation in lung damage, research focused on the control of the inflammatory process still remains a priority. 

In this context, more active research work is needed in order to define the ideal inflammatory pathway to target, as well as to identify and validate novel and noninvasive biomarkers useful in monitoring the inflammatory process and the therapeutic response. Furthermore, additional questions should be addressed to identify the patient phenotype most responsive to specific anti-inflammatory treatments in relation to patient age, genotype, CFTR modulator status, and timing of the start of treatment. As both the proinflammatory and proresolution arms of the inflammatory process contribute to the enhanced inflammatory responses in CF, the ideal anti-inflammatory treatment would need to effectively modulate both of these pathways.

In conclusion, we believe that the dysregulated inflammatory/immune response in CF will remain a key therapeutic target even in the postmodulator era, and that an intense research effort is still needed to develop safe and effective anti-inflammatory drugs for CF lung disease.
